# Supplementary material for: Drug repurposing for aging research using model organisms
Source: Aging Cell. 2017 Jun 16;16(5):1006–15. doi: 10.1111/acel.12626 (PMC5595691; doi:10.1111/acel.12626)
Supplement: Supplementary file 7 — Data S1 Zip‐Archive of all report cards. [file ACEL-16-1006-s007.zip › RC_VX6.pdf]

VX6

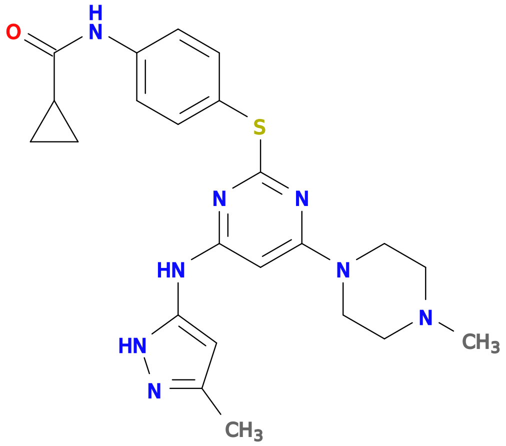

Database identifiers

ChEMBLCompound	CHEMBL572878
ZINC	ZINC13125756
eMolecules	31507548
eMolecules	36370217

Ranking

	Rank	Score
Drosophila	177/697	0.722
C. elegans	27/591	0.517

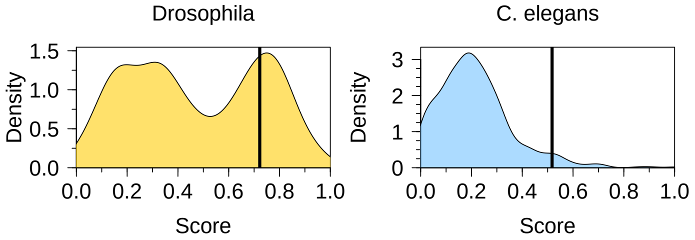

	Ageing implication	Domain conservation	Binding site conservation	Binding affinity	Bioavailability	Lipinski	Promiscuity	Purchasability	Drug approval	**Total**
Drosophila	0.792	0.937	1.0	0.932	(0.9)	0.0	-0.0	0.1	0.0	0.722
C. elegans	0.792	0.937	1.0	0.932	0.604	0.0	-0.0	0.1	0.0	0.517

Names

- N-4-4-(4-methyl-1-piperazinyl)-6-(5-methyl-1H-pyrazol-3-yl)amino-2-pyrimidinylthiophenylcyclopropanecarboxamid
- N-4-4-(4-methylpiperazin-1-yl)-6-(5-methyl-1H-pyrazol-3-yl)aminopyrimidin-2-ylsulfanylphenylcyclopropanecarboxa
- N-4-4-(4-methylpiperazin-1-yl)-6-(5-methyl-1H-pyrazol-3-yl)aminopyrimidin-2-ylthiophenylcyclopropanecarboxamid
- Tozasertib

Roles

ChEBI entry None has no roles

Status

Approved drug (according to ChEMBL)	No
Number of Rule of 5 violations	0
Binding affinity to original target in log units (RF-Score prediction)	7.62
Burns *C. elegans* bioavailability prediction	4.04

Compound Target Characteristics

Aurora kinase B

Best gene implication in ageing for this target family came from gene O55099 annotated in UniProt release 2014_02. Annotation GO 7568 (aging) was Inferred from Expression Pattern

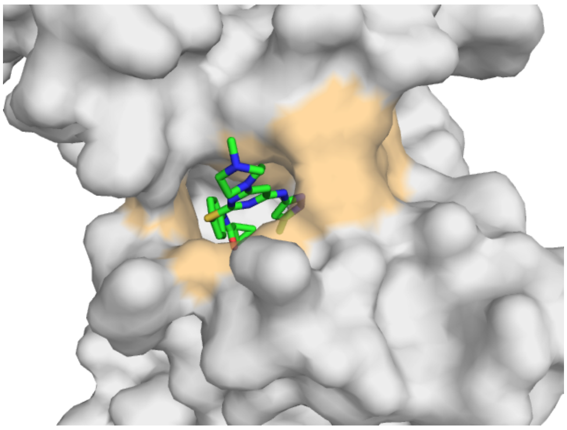

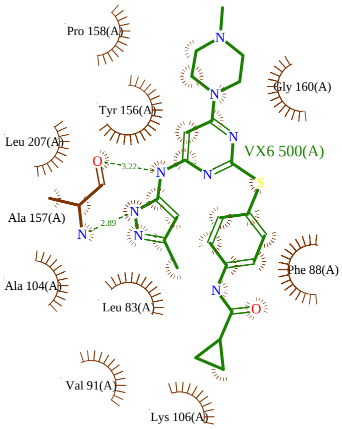

protein	amino acids contacts (binding site)
PDB:4af3:chainA:Q96GD4	L F V A K E Y A P G L
tr:J3QR41:J3QR41_HUMAN	L F V A K E Y A P G L
tr:J3KTD6:J3KTD6_HUMAN	L F V A K E Y A P G L
tr:J3QLN8:J3QLN8_HUMAN	L F V A K E Y A P G L
sp:Q96GD4:AURKB_HUMAN	L F V A K E Y A P G L
sp:O55099:AURKB_RAT	L F V A K E Y A P G L
sp:O70126:AURKB_MOUSE	L F V A K E Y A P G L
tr:Q9VGF9:Q9VGF9_DROME	L F V A K E Y A P G L
tr:Q27393:Q27393_DROME	L F V A K E Y A P G L
sp:O01427:AIR2_CAEEL	L F V A K E Y A P G L
sp:P38991:IPL1_YEAST	L F V A K E Y L V G L

	whole protein		domain-based		contact-based	
protein	ident	simil	ident	simil	ident	simil
PDB:4af3:chainA:Q96GD4	1.0	1.0	1.0	1.0	1.0	1.0
tr:J3QR41:J3QR41_HUMAN	0.5	0.5	0.54	0.54	1.0	1.0
tr:J3KTD6:J3KTD6_HUMAN	0.66	0.66	0.6	0.6	1.0	1.0
tr:J3QLN8:J3QLN8_HUMAN	0.66	0.66	0.76	0.76	1.0	1.0
sp:Q96GD4:AURKB_HUMAN	1.0	1.0	1.0	1.0	1.0	1.0
sp:O55099:AURKB_RAT	0.84	0.93	0.92	0.98	1.0	1.0
sp:O70126:AURKB_MOUSE	0.83	0.93	0.91	0.97	1.0	1.0
tr:Q9VGF9:Q9VGF9_DROME	0.41	0.65	0.61	0.87	1.0	1.0
tr:Q27393:Q27393_DROME	0.4	0.64	0.61	0.87	1.0	1.0
sp:O01427:AIR2_CAEEL	0.49	0.72	0.63	0.87	1.0	1.0
sp:P38991:IPL1_YEAST	0.36	0.69	0.47	0.83	0.82	0.85

aur (FBgn0000147) associated phenotypes

endomitotic cell cycle defective, lethal - all die before end of pupal stage, lethal - all die during P-stage, maternal effect, mitotic cell cycle defective, neuroanatomy defective, partially lethal - majority die, somatic clone, some die during pharate adult stage, some die during pupal stage, sterile

(Information from FlyBase)

air-2 (WBGene00000099) associated phenotypes
embryonic lethal, mitotic chromosome segregation variant, protein phosphorylation reduced, protein subcellular localization variant, sterile, tumorous germline

(Information from WormBase)

air-2 (UniProt:O01427) annotation
Function: Serine/threonine-protein kinase which mediates both meiotic and mitotic chromosome segregation. Required for histone H3 'Ser-10' phosphorylation (PubMed:9852156, PubMed:10354474, PubMed:10975519, PubMed:11050384, PubMed:11050385, PubMed:10983970, PubMed:12015116, PubMed:11940606). Phosphorylates tlk-1 at 'Ser-634', which enhances its activity (PubMed:15916946). Phosphorylates zen-4 at 'Ser-680'(PubMed:15854913). Required for the recruitment of bub-1 to the ring-shaped domain between chromosomes during meiotic anaphase I (PubMed:20729837). (PubMed:10354474, PubMed:10975519, PubMed:10983970, PubMed:11050384, PubMed:11050385, PubMed:11940606, PubMed:12015116, PubMed:15854913, PubMed:15916946, PubMed:20729837, PubMed:9852156).
Subunit: Interacts with zen-4 and icp-1. Part of a complexcontaining at least air-2; icp-1; csc-1 and bir-1. Interacts with tlk-1 and bmk-1. (PubMed:11050384, PubMed:11050385, PubMed:12707312, PubMed:15548597, PubMed:15916946).
Subcellular location: Cytoplasm, cytoskeleton (PubMed:9852156). Chromosome (PubMed:25475837, PubMed:9852156). Midbody (PubMed:9852156). Cytoplasm, cytoskeleton, spindle. Note=Meiotic and mitotic chromosomes (PubMed:9852156). During each division, relocates to the midbody microtubules (PubMed:9852156). Locates on chromosomes during metaphase and on the central spindle during anaphase. (PubMed:25475837, PubMed:9852156).
Developmental stage: Present during gametogenesis and throughout embryogenesis (at protein level). (PubMed:9852156).

(Information from UniProt)
